# Infectious Agents in Multiple Sclerosis: Viral Triggers, Antibody-Mediated Autoimmunity, and Parasitic Immunomodulation

**DOI:** 10.3390/biom16060899

**Published:** 2026-06-18

**Authors:** Dafni F. T. Frohman, Stella E. Tsirka

**Affiliations:** 1 Department of Neurology, Mount Sinai Morningside/West, New York, NY 10019, USA; 2 Department of Pharmacological Sciences, Renaissance School of Medicine, Stony Brook University, Stony Brook, NY 11794, USA

**Keywords:** multiple sclerosis, Epstein–Barr virus, molecular mimicry, antibodies, helminths, immune regulation, hygiene hypothesis, neuroinflammation

## Abstract

Multiple sclerosis (MS) is a chronic immune-mediated disease of the central nervous system characterized by demyelination, neuroinflammation, and progressive neurodegeneration. While there is a small component of genetic susceptibility to MS risk, environmental factors, including infectious exposures, are gaining increased recognition as playing a critical role in MS initiation and progression. Viral infections, especially by Epstein–Barr virus (EBV), have emerged as strong candidates and triggers of MS symptoms, through antibody-mediated molecular mimicry and B-cell dysregulation. In contrast, parasitic infections, including helminths and select protozoa, appear to exert neuroprotective effects by skewing immune responses toward regulation and tolerance. In this review, we examine antibody-driven mechanisms by which viral pathogens promote autoimmunity in MS and contrast these with parasite-induced immunoregulatory pathways that suppress pathogenic inflammation. We further discuss diagnostic and therapeutic implications, highlighting how insights from infectious immunology may inform novel strategies for MS treatment.

## 1. Introduction

In autoimmune diseases, the immune system fails to recognize specific self-antigens and becomes hyperactive. This causes increased immune response against oneself, with resultant inflammation and tissue destruction [1]. Autoimmune disorders can affect multiple organ systems, such as in the case of systemic lupus erythematosus (SLE), or can be limited predominantly to one organ system, such as in the case of multiple sclerosis (MS), which affects the central nervous system (CNS). MS is the most common autoimmune disease of the CNS and is usually diagnosed between the ages of 20 and 50 years. MS predominantly affects women, with an incidence ratio of 3:1 over men [2,3]. According to the National Multiple Sclerosis Society, approximately 1 million people in the United States are affected by MS, and more than 2.9 million people are living with MS worldwide. It is characterized by immune-mediated demyelination, axonal injury, and neurodegeneration [4,5]. Pathological hallmarks include inflammatory demyelinating plaques, oligodendrocyte loss, and gliosis. CD4^+^ T helper subsets—particularly T helper 1 (Th1) and 17 (Th17) cells—are thought to drive CNS inflammation, while CD8^+^ T cells and autoreactive B cells contribute to myelin destruction and axonal degeneration [4,6,7,8,9]. Normally, during antibody production, B cells undergo somatic hypermutation to generate new antibodies against target antigens. In MS, during this somatic hypermutation, B cells produce self-targeting antibodies, leading to high levels of CNS inflammation and loss of myelin [10,11].

Clinically, MS has been classified into relapsing–remitting (RRMS, experienced by about 80% of people with MS), primary progressive (PPMS, experienced by ~15% of people with MS), and secondary progressive (SPMS) subtypes [5]. RRMS is characterized by episodes of sudden neurological dysfunction, such as blurry vision or vision loss, fatigue, numbness, weakness, or postural instability that resolves after the acute event [12]. PPMS presents similarly, but with steady, gradual worsening of neurological function rather than resolution of symptoms [13]. SPMS occurs when the patient begins with having the RRMS subtype, with eventual transformation to worsening neurological function and disability [14]. However, increasing evidence suggests that these phenotypes represent a disease continuum [15] rather than strictly distinct subtypes and have overlapping immunopathological mechanisms that evolve over time.

Despite extensive research, the etiology of MS remains incompletely understood. The rising incidence of MS over the past two centuries [16] suggests a major contribution of environmental factors. Epidemiological data demonstrate a strong latitudinal gradient, with higher MS prevalence at greater distances from the equator—an observation linked to reduced ultraviolet exposure and vitamin D deficiency [5]. People who have moved farther from the equator before age 15 take on the geographical risk of their host location rather than where they were born. Conversely, people who moved after the age of 15 years to a place farther from the equator have the protective influence of their original country [17]. Additional environmental risk modifiers include smoking, diet, and prior infections, particularly those occurring in childhood or adolescence [5].

One hypothesis that has been introduced to help explain the increase in incidence of autoimmune disorders is the *Old Friends Hypothesis*, which emerged in the early 2000s [18,19,20]. It posits that exposure to evolutionarily conserved microorganisms and parasites is required for appropriate immune education, as microbial inputs drive the immune system. Conversely, lower exposure to pathogens early in life may contribute to greater risk of immune-mediated diseases later in life [4]. In particular, it notes that humans have co-evolved with the microbiota in their intestines. These microbiota are no longer able to function outside of our gut. Humans, in turn, gain much information from the microbiota and eventually develop a diverse immune system, allowing us to more easily recognize new pathogens. Each person’s intestinal microbiota is unique, having adapted to their personalized environment. The *Old Friends hypothesis* is strengthened by observational studies, such as one that showed that children exposed to older siblings and daycare centers were less likely to have frequent infections, especially when their exposure was before the age of 3 years old [20,21]. That observation was hypothesized to be related to the increased pathogen exposure those children had through their environment. Other studies have also found a strong inverse relationship between having at least two older siblings and developing multiple sclerosis [21]. While neither of these studies can show causality, this association has been found repeatedly.

**Figure 1:**
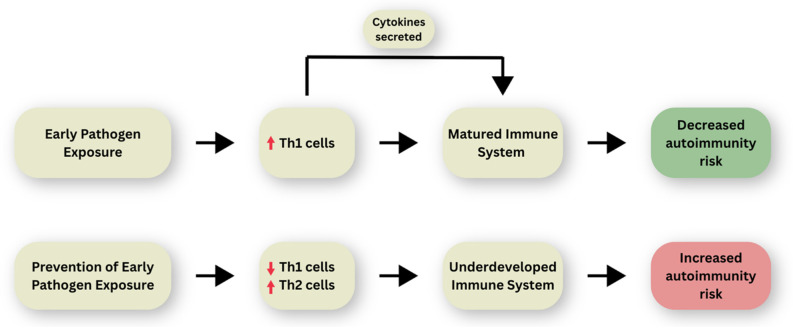
Schematic depicting the Hygiene Hypothesis. In normal early exposure to various pathogens, the immune system develops through the activation of T helper cells 1 (Th1), which secrete cytokines that further support immune cell maturation, lowering the risk for generation of antibodies against self-antigens and the risk of autoimmune disorders (**top row**). However, limited exposure to early pathogens supports activation of Th2 cells (at the expense of Th1) and the secretion of Th2-related cytokines. This leads to an underdeveloped immune system and the uncontrolled generation of antibodies by B cells, including antibodies against self-antigens, thus increasing the risk of autoimmunity (**bottom row**).

A similar hypothesis known as the *Hygiene Hypothesis*, adapted from the *Old Friend Hypothesis*, further posits that modern sanitation, reduced pathogen exposure, and increased societal cleanliness create an environment that fails to generate the appropriate stimuli to educate and regulate the human immune system (Figure 1). As a result, the human immune responses skew toward pro-inflammatory and autoreactive pathways [4,22]. On a microscopic level, the *Hygiene Hypothesis* postulates that infections by bacteria and protozoa activate Th1 cells and lead to the secretion of cytokines, maturing the immune system. Decreased contact with infectious agents results in decreased Th1 activity and a compensatory increase of T helper 2 (Th2) cells. Prevention of infections during childhood, therefore, is associated with a predominance in Th2 cell activity and underdevelopment of the immune system, making it vulnerable to new pathogens not recognized by the body [23].

Autoimmune disorders usually present with an increase in Th1 activity rather than a decrease; thus, the pathophysiology has not yet been fully understood [23]. In this context, understanding how specific infectious agents influence MS risk is of critical importance. This review paper aims to discuss antibody-mediated mechanisms derived from viruses and parasites that contribute to the likelihood of developing MS and how these processes modify the progression of the disease. We also aim to review current literature surrounding possible treatments through helminth-derived therapies and other emerging care options.

## 2. Viral Antibodies as Drivers of Multiple Sclerosis Pathogenesis

### 2.1. Epstein–Barr Virus and MS Risk

Human Herpes Viruses (HHV) infect most individuals early in life and persist in a latent state. Over 90% of adults have been infected with at least 1 species of HHV, typically within the first decades of their life [20]. Once within the body, this family of viruses is known for remaining latent in B-cell memory but may undergo periodic reactivation [20,24]. Among them, the Epstein–Barr virus (EBV; HHV-4) has the strongest and most consistent association with MS diagnosis. EBV is a pathogen that is present in nearly 90% of all adults [25,26], and most EBV infections occur at a young age. The virus is typically passed through the saliva of infected individuals and can establish an infection within B cells through gp350/220 attachment to the complement receptor CD21 or endocytosis by Neuropilin 1/NRP1 on the cell surface [26]. To evade the immune system, EBV remains in a restricted latent stage but is detectable by the expression of EBNA-1 (EBV Nuclear Antigen 1), EBER (EBV encoded RNAs), and BART (BamHI A Encoded Transcripts). Although the functions of EBER and BART transcripts are not fully understood, EBNA1 has been thought to support EBV replication during B cell division. Infected individuals develop anti-EBNA1 antibodies against infected cells. Although primary EBV infection may often be asymptomatic, it can also cause infectious mononucleosis (IM), a relatively common infection spread through saliva, which is associated with a markedly increased risk of cancer and MS [1,25].

Large longitudinal studies have demonstrated that EBV infection almost invariably precedes MS onset [25,27]. For example, Bjornevik et al. in 2022 [27] analyzed serum antibodies of EBV from 800 individuals with MS among over 10 million people in the US army over a 20-year period from 1993 to 2013. They reported that while 35 of the ~800 people with MS were initially EBV-seronegative at the start of the study, 34 of the 35 people became infected with the virus before the onset of their MS [27]. The rest of the 800 participants were found to already have a prior EBV infection [27]. In a different study, it was noted that for individuals infected with EBV, anti-EBV antibodies (especially against EBNA-1) significantly increased over the five years prior to development of MS or any neurological symptoms [1,24], suggesting that EBV-driven immune responses may contribute to early disease pathogenesis or at least be associated with a higher risk of development of MS [1]. That being said, no clear causality at this time has been established, and although a temporal pattern has been shown, all these studies only demonstrate associations between EBV infection and MS development.

### 2.2. Molecular Mimicry and Cross-Reactive Antibodies

**Figure 2:**
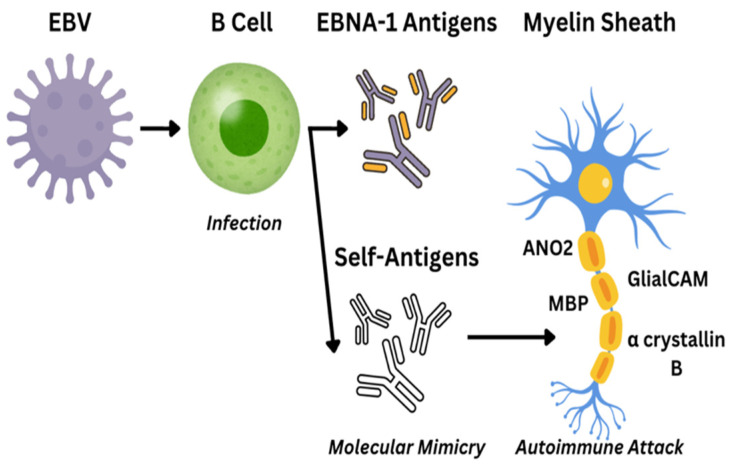
Schematic of EBV infection and the production of self-antigens through molecular mimicry. B cells generate antibodies against EBV proteins, including against EBNA-1, which has different regions that are homologous to sequences on the myelin protein MBP, or on GlialCAM and a few other human proteins. Through molecular mimicry, antibodies against EBNA-1 also end up recognizing these human proteins and thus attack myelin, eventually resulting in autoimmune MS.

One of the most compelling mechanisms linking EBV to MS is *molecular mimicry*, a concept recognizing that there may be similarity in the structural homology of a pathogen and self-proteins, creating either a way for a pathogen to evade the human immune system or an increased risk of autoreactivity as the body attempts to respond to the pathogen through T- and B-cell activation [28,29]. EBNA-1 contains multiple linear amino acid sequences that closely resemble CNS proteins critical for myelin integrity and neuronal function [1]. Within the 47-amino-acid stretch of EBNA-1, there are four distinct sequences that overlap with human proteins. Notably, it shares homology with myelin basic protein (MBP) [30], GlialCAM (Glial Cell Adhesion Molecule) [31], ANO2 (anoctamin-2) [32], and α-crystallin B [33,34]. MBP is the second most abundant protein of the myelin sheath; GlialCAM localizes closely to the nodes of Ranvier; ANO2 is involved with electrical conduction along myelinated axons; and alpha crystallin B serves as a checkpoint against neuroinflammation. Antibodies generated against EBNA-1 may therefore cross-react with these proteins, leading to immune-mediated damage of myelin and axons (Figure 2). One study, by Holmøy et al., supported this proposed mechanism by showing that CD4 ^+^ T cells isolated from the cerebrospinal fluid (CSF) of patients with MS were specific not just for EBV peptides but also many recognized MBP peptides [35]. Moreover, in a recent animal study by Maguire et al., even more sites of molecular mimicry were denoted between EBV cells and those of people with MS, suggesting that there are additional sites that increase the autoreactivity of the disease [29]. These results amplify the previous findings by Zamecnik et al., who reported that ~8% of the MS cases had an auto-antibody profile deriving from EBV mimicry [36].

Molecular mimicry has also been described for other autoimmune diseases associated with resolution of infections, such as rheumatic fever following Group A Streptococcus (GAS) infection, in which the M protein in GAS mimics myosin. Antibodies formed in response to the M protein cross-react with myosin, leading to cardiac myositis and valvulitis [37]. In MS, similarly, cross-reactive antibodies may initiate or amplify CNS inflammation by promoting complement activation, antibody-dependent cellular cytotoxicity, and epitope spreading [1,38]. Following the recognition of myelin antigens through molecular mimicry, it is possible that additional autoantibodies are generated against neighboring epitopes through *epitope spreading*. Epitope spreading, the recognition by B and T cells of many epitopes or new antigens beyond the epitope on the original antigen, was first recognized in the experimental autoimmune encephalomyelitis (EAE) animal model of MS [39]. There, it was reported that the antigenic targets of the antibodies were not just myelin oligodendrocyte glycoprotein (MOG) that had been used to induce EAE; rather, the epitope targets had expanded and diversified during progression of the EAE to neighboring regions of the DNA sequence [40].

### 2.3. Clinical Cases of Viral Infections and MS Diagnosis

In addition to EBV, other viruses have also been studied to assess associations with increased MS risk. During the COVID-19 pandemic, there were reports of SARS-CoV-2 infections resulting in CNS demyelinating disease, diagnosed as MS based on imaging data, or relapses of the disease [41,42]. A study looking at meta-analyses of linkages between MS and viral infections found that SARS-CoV-2 had a greater odds ratio (OR = 3.74) than EBV (OR = 3.33) of being associated with MS [43]. Alongside these two viral infections, there were increased odds ratios associated with HHV-6 (OR = 2.81), HHV-8 (OR = 2.42), Herpes Simplex Virus-2 (OR = 1.76), and Varicella Zoster Virus (OR = 1.71). The authors hypothesized that each of the viral pathogens induces MS in distinct ways, all of which may be through increased inflammation in the CNS; though, again, these molecular/cellular pathways have not been clearly deciphered at this time [43].

A recent study aimed to examine common factors between COVID-19 and MS, using single-cell RNA sequencing (scRNA-Seq) with genome-wide association studies (GWAS) and quantitative trait loci (QTL) analyses from patients with the two diseases. The study revealed that the genes *DR1*, *IKZF3*, and *RUVBL2* were risk factors for both MS and COVID-19 development, whereas the gene *ANAPC5* was characterized as a protective factor against both. This finding suggests a possible interaction between the two diseases and implicates that they share common inflammatory mechanisms, with the caveat that the study included blood samples from only 5 MS patients, 12 COVID-19 patients, and 6 controls [44]. This was also corroborated by a study that examined cytokine profiles in patients with MS prior to 2020 and those who had their first MS symptoms during the COVID-19 pandemic, i.e. between 2020 and 2021, effectively assessing whether a general large-scale viral exposure influenced MS immunopathogenesis. Sampling from 20 pre-pandemic MS cases, 36 new-onset cases during the pandemic, and 20 controls (other non-inflammatory neurological disease), the study reported that in the new-onset MS cases during the pandemic, there was an increase in the activation of peripheral cytokines, including in B cell recruitment and survival (CXCL13 and BAFF) [45], suggesting again that the exposure to the SARS-CoV-2 inflammatory landscape may have sensitized patients to MS. No molecular mimicry pathways have been found at this time more directly linking EBV and COVID-19. Overall, the data associating other viruses with MS are much less clear and understood relative to the correlation of MS with EBV, as denoted previously.

### 2.4. B Cells, Genetic Susceptibility, and Incomplete Penetrance

T-cell-dependent B cell responses originate and take shape in germinal centers (GCs). In these specialized microenvironments, follicular T helper (T_FH_) cells regulate B cell activation and their differentiation into memory B cells. Memory B cells can secrete Ig as well as pro- and anti-inflammatory cytokines. Aberrant B cell responses are prevented, at least in part, by follicular regulatory T (T_FR_) cells, which suppress GC-derived autoreactive B cell responses through the expression of inhibitory receptors and cytokines [46]. During MS and other autoimmune diseases, B cells expand in sites of chronic inflammation, e.g., around the meninges. These additional sites of B cell expansion are called Ectopic Lymphoid Follicles (eLF) and have been viewed as reservoirs of autoreactive B cells and of T_FH_ [46,47], and they notably lack T_FR_ cells [48]. In several reports in the literature, MS progression and severity of symptoms has been correlated with the presence of eLFs [49,50,51], which contain both B cells and Th17 cells, leading to the generation and maintenance of a pool of pro-inflammatory cytokines. This, in turn, results in T cell reactivation and B cell differentiation [52].

EBV’s tropism for B cells further implicates humoral immunity in MS pathogenesis. EBV-infected memory B cells can persist within the CNS, act as potent antigen-presenting cells, and produce autoreactive antibodies [53]. The strong efficacy of B-cell-depleting therapies in MS underscores the importance of this pathway.

However, EBV infection alone is insufficient to cause MS, as only a small fraction of infected individuals develop disease. Genetic susceptibility—particularly the HLA-DRB1*15:01 allele—appears to interact with EBV-specific immune responses to increase MS risk [54]. In a study performed by a team at Stanford University and the Karolinska Institute, 650 patients with MS were compared with matched controls. The investigators reported that antibodies to the EBNA-1 antigen were increased in patients with MS, especially in patients who carried the risk allele HLA-DB1*15:01, postulating an independent risk factor for developing the disease, though still not completely explaining the penetrance. They theorized that other environmental factors, such as tobacco use, Vitamin D levels, and geographic environment, may also further modulate MS susceptibility and progression [54].

## 3. Parasitic Infections as Immunomodulatory Protectors in MS

### 3.1. Helminths and Immune Regulation

In contrast to viral infections, parasitic infections—especially helminths—have been associated with reduced MS risk and milder disease progression [55,56,57,58]. Helminths are multicellular parasitic worms that have co-evolved with humans for thousands of years and remain prevalent in many regions of the world. They have immune-modulatory abilities and exert their actions both on the innate and adaptive immune system [4]. They can be as small as millimeters or as long as meters in length [24]. It is estimated that approximately one third of all humanity presently has helminths, a decrease from what was nearly universal before the 20th century [59].

Helminths exert powerful immunomodulatory effects, promoting a Th2-biased immune response characterized by increased production of cytokines IL-4, IL-5, IL-10, and TGF-β [60]. These cytokines suppress pro-inflammatory Th1 and Th17 responses, which are central to MS pathogenesis, as previously denoted. Epidemiological studies reveal an inverse correlation between helminth endemicity and MS prevalence, supporting a protective association at the population level.

### 3.2. Regulatory T and B Cells in Parasite-Mediated Protection

Helminth infections induce the expansion of regulatory T cells (Tregs), particularly CD4^+^CD25^+^FoxP3^+^ cells, which suppress autoreactive immune responses through secretion of IL-10 and TGF-β. In addition, regulatory B cells (Bregs), induced during parasitic infection [61], contribute to immune tolerance by producing IL-10 and dampening pathogenic T-cell activity, as has been shown in other pathologies [62,63].

Early epidemiological studies have reported that schistosomiasis, caused by *Schistosoma*, and MS are mutually exclusive worldwide [64], suggesting that this parasitic infection may offer protection against MS. Similarly, the prevalence of MS appears to fall steeply once a critical threshold of *T. trichiura* prevalence (about 10%) is exceeded in a population [65]. Clinical observations indicate that MS patients with natural helminth infections experience fewer relapses, have fewer MRI-imaged demyelinating lesions, and have slower disability progression. Results of the HINT-2 clinical trials demonstrated that *Trichuris suis* ova (TSO) given at a dose of 2500 TSO every 2 weeks over a 10-month period resulted in a mild increase of eosinophils, *T. suis*-specific antibodies [66], and the expansion of circulating IgG- and IgA-switched memory B cells. These increases were accompanied by an increase in CD4^+^CD127^−^CD25^high^ Tregs in TSO-treated patients, compared with the amount in placebo-treated MS patients [66,67]. Chronic parasitic infections have been reported to shift Th1/Th17 responses to Th2/Tregs, reducing Th1 cytokines (IFN-γ, TNF-α) and increasing Th2/Tregs cytokines (IL-4, IL-5, TGF-β) [68,69]. Moreso, removal of the parasitic infection through anti-helminthic treatment may lead to increased MS activity [70].

However, other experiments with the anti-parasitic drug ivermectin in the Experimental Autoimmune Encephalomyelitis (EAE) animal model of MS resulted in decreased EAE/MS symptoms, as ivermectin prevented the infiltration of inflammatory cells into the CNS. Specifically, it promoted immunosuppressive Treg cells and inhibited pro-inflammatory Th1 and Th17 cells by decreasing IFN-γ and IL-17A production and increasing IL-2 levels, expression of CD25, and phosphorylation of STAT5 in the CNS [71]. These contradictory reports, as far as outcomes are concerned, provide further evidence that it is critical to define the molecular and cellular pathways affected by parasitic infections, as they have not yet been clearly established or understood.

### 3.3. Protozoan Infections and Neuroimmune Modulation

Certain protozoan infections, such as chronic *Toxoplasma gondii* infection, have also been inversely associated with MS. Although *T. gondii* induces a strong initial Th1 response, chronic infection promotes compensatory regulatory mechanisms, including increased IL-10 and IL-27 production and expansion of Tregs [55,72]. Exposure to parasites, especially at a young age, has been associated with lower risk of developing MS [72]. EAE animals infected chronically with *T. gondii* exhibited a switch towards suppression of Th17 responses, an increase in Treg-produced cytokines, a reduction in blood–brain barrier permeability, and an amelioration of the demyelination status [72].

## 4. Pathogenic vs. Protective Antibody Responses in MS

**Figure 3:**
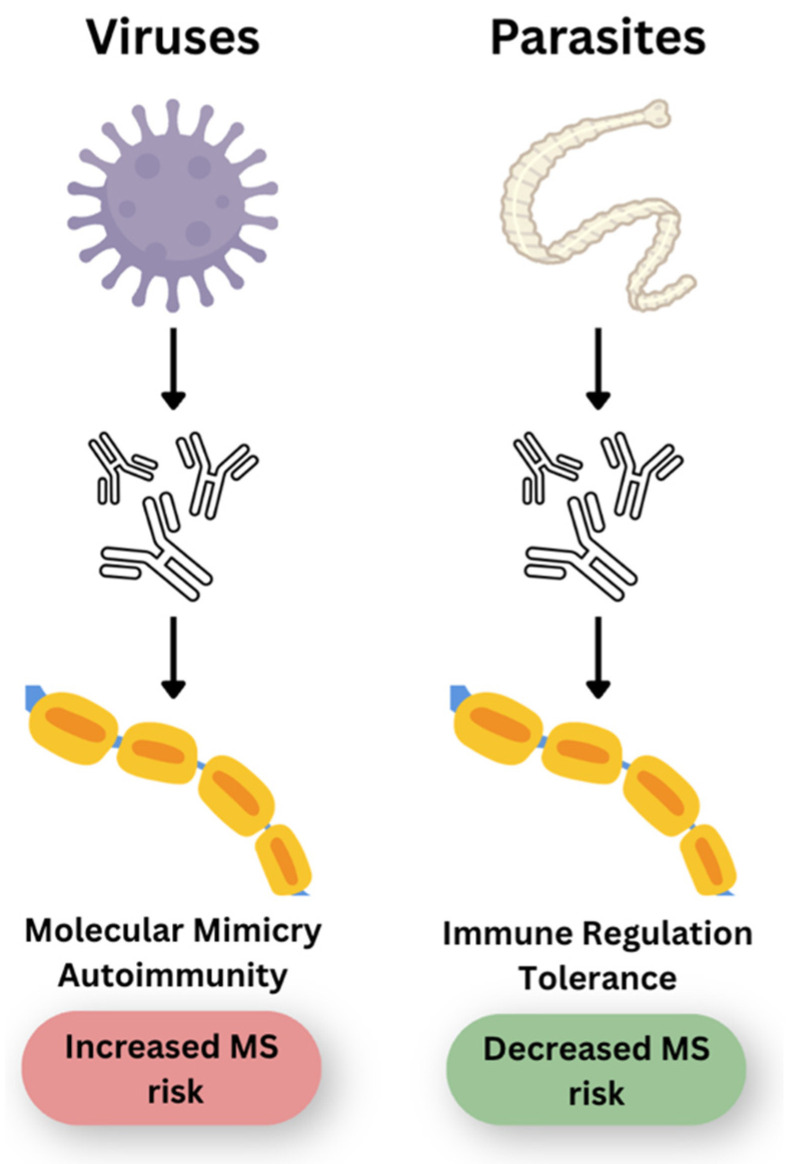
Schematic of Viral Infections and Parasitic Infections, with their Theorized Impact on the Human Immune System. Viral infections, especially EBV, result through molecular mimicry and subsequent epitope spreading in the generation of antibodies against myelin self-antigens, leading to autoimmunity and increasing the risk of MS. Parasitic infections, on the other hand, have been associated with the regulation of immune tolerance and a decrease in risk of developing MS.

Viral and parasitic infections may exert opposing effects on immune homeostasis in MS. Infections from viruses such as EBV promote immune activation, autoreactive antibody production, and epitope spreading, resulting in chronic CNS inflammation. In contrast, parasitic infections induce regulatory immune networks that mitigate inflammation, improve MS symptoms, and preserve tolerance (Figure 3).

It is possible that these studies and epidemiological correlations point towards the idea that, in MS, it is the specificity of the antibodies rather than a general inflammatory state that determines pathogenicity. Antibodies targeting myelin and proteins on or close to the nodes of Ranvier may contribute to disease progression, whereas parasite-induced antibodies participate in immune regulation without having associated concurrent tissue damage.

## 5. Diagnostic and Therapeutic Implications

The presence of intrathecal markers, reflected by the detection of oligoclonal bands in cerebrospinal fluid (CSF) and/or CNS proteins (e.g., Glial Fibrillary Acidic Protein [GFAP], Kappa free light chain index [kFLC], or Neurofilament Light chain [NfL]), along with neuroimaging, remain key diagnostic features of MS, along with obtaining a related history in speaking to the patient. Analysis of protein levels of IgA, IgM, IgG, and albumin in the serum and in the CSF samples of 80 patients with MS and 28 matched controls revealed that in all types of MS, IgM and IgG indices were increased in the CSF compared to the control group. More, people with MS were noted to have an increased presence of IgM+ and IgG+ B cells in the leptomeninges (accompanied by high responsiveness and activation of neighboring cortical microglia). The high IgM and IgG indices correlated significantly with NfL levels, but not with clinical or radiological parameters of the disease [73]. The intrathecal concentration of IgA was not different between groups.

The role of these intrathecally produced antibodies remains unclear. There are reports indicating that treatments targeting depletion of B cells are effective in attenuating MS symptoms, but not in decreasing intrathecal IgG antibodies [73]. In the case of EBV, intrathecally synthesized EBV antibodies were detectable in 26% of pediatric and 10% of adult-onset MS patients [74,75]. This paradox of high levels of serum-detectable EBV antibodies versus the low presence of antibodies in the CSF can be explained by the hypothesis that B cells responsible for intrathecal antibody production are primed during and through acute EBV infection to subsequently enter the CNS of patients with MS [75]. Another hypothesis is that EBV induces immune evasion by blocking the activation of T cells, which then cannot stimulate B cells to produce antibodies. Expanded T lymphocyte clones in CSF appear to be specific for EBV-infected B cells. Nevertheless, the immune evasion appears to be directed at EBV-infected cells rather than the actual virus [76]. A vaccine against EBV could prevent EBV and also be directed against EBV-infected cells [77]. From a therapeutic perspective, parasite-derived immunomodulatory molecules represent an appealing avenue for treatment development. While clinical trials involving controlled exposure to parasites (Live Hookworm Larvae) have been limited by side effects (e.g., the WIRMS-1 trial NCT00630383) [78,79], preclinical studies using defined helminth antigens or molecules demonstrate strong immunoregulatory potential [70]. Established treatments for MS, such as IFN-β, have already been shown to have antiviral properties, especially against EBV [80]. Clinical interventions targeting the management of EBV through infusion with autologous EBV-specific T cells at a first clinical episode of MS, or using Truvada (tenofovir/emtricitabine, an antiviral against EBV, which may reduce EBV levels in saliva and blood of MS patients), are potential therapies for MS [29,81,82]. However, consensus has not yet been established about viral pathogenesis in MS, and some parasitic infections can have adverse gastrointestinal effects [83,84]. Identifying safe, targeted immunomodulators inspired by parasitic biology may offer novel strategies to restore immune balance in MS.

## 6. Conclusions and Future Directions

Compelling evidence implicates viruses as major environmental triggers for MS through antibody-mediated molecular mimicry and B-cell dysregulation. As the CNS is continuously surveyed by virus-specific T cells, which are thought to protect against neurotropic viruses that may reactivate, it is possible that the presence of these cells can lead to dysregulation of self-tolerance and induce B and T cell subsets, thus triggering MS symptoms. On the other hand, parasitic infections appear to be protective against MS by promoting immune regulation and tolerance. Together, these findings may give support to the *Old Friends* hypothesis and emphasize the importance of immune education early in life.

Future studies should focus on longitudinal analyses of infectious exposure and deeper characterization of pathogenic versus regulatory antibody responses. An EBV vaccine could also prevent EBV from developing in future generations, a more direct way of mitigating MS risk. Moreover, development of parasite-inspired immunotherapies could prove to be beneficial for MS patients. Integrating insights from infectious immunology may ultimately lead to innovative approaches for preventing and treating MS.

## Data Availability

No new data were created or analyzed in this study. Data sharing is not applicable to this article.
